# Engineered Accumulation of Protocatechuate in Corn Biomass to Enhance Biomanufacturing

**DOI:** 10.1021/acssuschemeng.5c09025

**Published:** 2025-11-11

**Authors:** Yang Tian, Bumkyu Kim, Irem Pamukçu, Emine Akyuz Turumtay, Alexis H. Tan, Victoria Saini, Ariana Irma Chavez, Anna Tang, Anna Z. Su, Edward E. K. Baidoo, Jorge Rencoret, José C. del Río, Timothy J. Donohue, Daniel R. Noguera, Aymerick Eudes

**Affiliations:** 1 Joint BioEnergy Institute, Emeryville, California 94608, United States; 2 Environmental Genomics and Systems Biology Division, Lawrence Berkeley National Laboratory, Berkeley, California 94720, United States; 3 Department of Civil and Environmental Engineering, 5228 University of Wisconsin-Madison, Madison, Wisconsin 53706, United States; 4 Wisconsin Energy Institute, 5228 University of Wisconsin-Madison, Madison, Wisconsin 53726, United States; 5 Great Lakes Bioenergy Research Center, 5228 University of Wisconsin-Madison, Madison, Wisconsin 53726, United States; 6 Biological Systems and Engineering Division, 1666 Lawrence Berkeley National Laboratory, Berkeley, California 94720, United States; 7 Instituto de Recursos Naturales y Agrobiología de Sevilla, IRNAS-CSIC, Avenida de la Reina Mercedes, 10, Sevilla 41012, Spain; 8 Department of Bacteriology, 5228 University of Wisconsin-Madison, Madison, Wisconsin 53706, United States

**Keywords:** corn stover, 3,4-dihydroxybenzoate, coproduct, biological funneling, 2-pyrone-4,6-dicarboxylate, *Novosphingobium aromaticivorans*

## Abstract

The in-planta accumulation of coproducts in crops can enhance the value of lignocellulosic biomass and facilitate a sustainable bioeconomy. Corn stover represents a major renewable source of lignocellulose for the production of advanced biofuels and bioproducts. In this study, we engineered corn with a bacterial gene encoding a dehydroshikimate dehydratase (QsuB) to overproduce protocatechuate (DHBA). Transgenic corn lines accumulate up to 2.9% DHBA on a dry weight basis in leaf and stem biomass. DHBA occurs in the form of glucosides that are extractable from biomass using aqueous methanol as the solvent. The analysis of lignin did not show any evidence for the incorporation of DHBA; however, an increase in the lignin syringyl to guaiacyl ratio and a higher relative abundance of *p*-coumarate groups compared with total lignin units were observed in QsuB-modified corn. Alkaline hydrolysates prepared from QsuB corn were enriched in DHBA compared to the hydrolysates obtained from wild-type biomass, which contained mostly *p*-coumarate and ferulate. Using engineered *Novosphingobium aromaticivorans* as a production host, a 375% improvement in 2-pyrone-4,6-dicarboxylate titers was achieved through biological upgrading of alkaline hydrolysates derived from QsuB corn compared to unmodified biomass. Our data demonstrate an engineering strategy to overproduce DHBA in corn that can facilitate sustainable manufacturing of other valuable bioproducts using stover as a feedstock.

## Graphical abstract

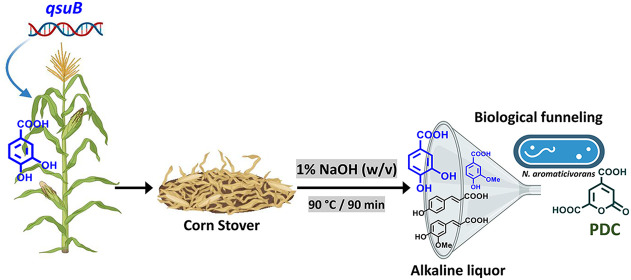

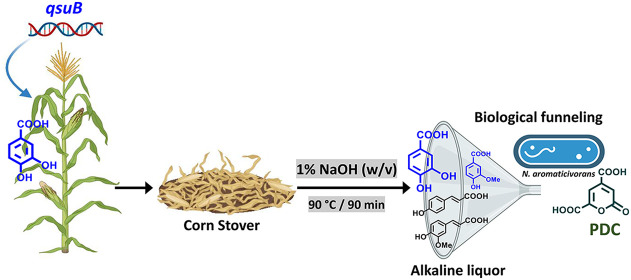

## Introduction

Lignocellulosic biomass serves as a crucial renewable resource for the production of advanced biofuels and bioproducts.^1^ However, the high cost associated with deconstructing plant biomass to sugars and aromatics remains one of the challenges preventing the deployment of economically sustainable biomanufacturing.^2^ The engineering of plants to accumulate value-added coproducts that can be recovered at biorefineries has been proposed as a strategy to provide additional revenue streams and improve the economics of advanced bioproducts.^3^ In this case, engineered bioenergy crops can provide polysaccharides for making advanced bioproducts and also accumulate valuable compounds such as polymers, platform chemicals, pharmaceuticals, flavors and fragrances that can be easily extracted from biomass, purified, and sold.^4^ Moreover, alkaline solvent treatment of lignocellulose can effectively release simple molecules like aromatics and aliphatic hydroxyacids from the lignin and hemicellulose in cell walls.^5^ These molecules can be further upgraded biologically into higher-value chemicals using engineered microbial strains such as *Pseudomonas putida*,^6,7^ *Novosphingobium aromaticivorans*,^8^ *Rhodosporidium toruloides*,^9,10^ *Rhodococcus opacus*, and *Sphingobium lignivorans*, which are capable of consuming a diverse set of organic carbon sources.^11^

In the United States, corn stover is the most abundant agricultural residue with a potential supply of ∼ 159 million dry tons per year of lignocellulosic biomass.^12,13^ However, there are few examples of metabolic engineering for the accumulation of valuable bioproducts in corn lignocellulosic biomass.^14,15^ In this study, we engineered corn to overproduce protocatechuate (i.e., 3,4-dihydroxybenzoate or DHBA), which is an aromatic monomer used for several pharmacological applications due to its antioxidant activity as well as anti-inflammatory, anticancer and antiviral properties.^16–19^ DHBA can also serve as a precursor to performance-advantaged food packaging materials,^20^ or used in fuel cell applications as an alternative to ascorbic acid.^21^ Furthermore, several microbial strains have been engineered to upgrade DHBA into platform chemicals including muconate,^22^ beta-ketoadipate,^23^ 2-pyrone-4,6-dicarboxylate,^24^ pyridine-2,4-dicarboxylate,^25^ polyhydroxyalkanoate,^26^ and other industrially relevant aromatics such as gallate^27^ and vanillin.^28^

While DHBA is present in various plant species, the precise metabolic pathways and genes responsible for its biosynthesis are yet to be discovered.^17^ We showed previously that DHBA can be overproduced in plants via heterologous expression of plastid-targeted bacterial 3-dehydroshikimate dehydratase (QsuB) to convert endogenous 3-dehydroshikimate into DHBA (Figure 1a).^29–32^ In this work, we transformed corn (*Zea mays*) with a DNA construct designed for *qsuB* expression and showed that these engineered line accumulate DHBA *in planta*. DHBA content ranged between 2.2–2.9 dwt% in transgenic lines grown in the greenhouse, which represents a 630- to 800-fold increase compared to titers measured in wild-type control plants. We also found that DHBA mainly occurs as glycoside conjugates in biomass, which can be readily extracted with aqueous methanol solvent. The analysis of cell walls showed only trace amounts of ester-linked DHBA, while no DHBA could be detected in isolated lignin fractions. Aromatics extracted from QsuB corn biomass by dilute alkali were greatly enriched in DHBA. We also found that higher production of the polyester precursor 2-pyrone-4,6-dicarboxylate (PDC) was achieved after biological upgrading of aromatics derived from QsuB corn biomass using engineered *N. aromaticivorans* as a production host. Thus, introducing the DHBA coproduct trait into corn, through QsuB expression, offers a dual benefit: enhancing corn stover’s value as a bioenergy feedstock and enabling the production of advanced bioproducts.

**Figure 1:**
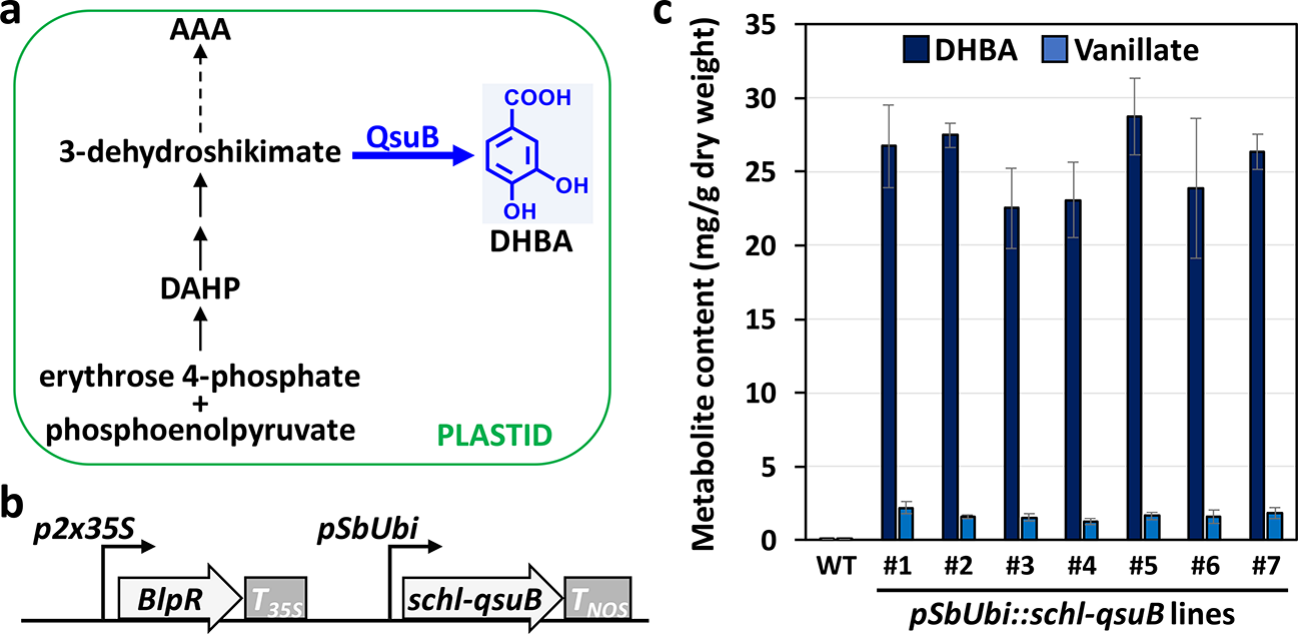
Production of DHBA in corn. (a) Schematic diagram of the conversion of 3-dehydroshikimate into DHBA catalyzed by plastid-targeted QsuB. AAA, Aromatic amino acids; DAHP, 3-deoxy-d-arabinoheptulosonate 7-phosphate. (b) Construct used for corn transformation. The T-DNA contains a selection marker for glufosinate resistance (*BlpR*) under the control of a duplicated 35S promoter (*p2×35S*) from cauliflower mosaic virus (CaMV) and harbors the *qsuB* gene sequence under the control of the promoter of a sorghum polyubiquitin gene (*pSbUbi*). *T*_*35S*_ and *T*_*NOS*_ denote the terminators from the CaMV 35S and *Agrobacterium tumefaciens* nopaline synthase genes, respectively. *Schl* encodes a plastid signal peptide. (c) DHBA and vanillate contents in seven independent transgenic lines. Values are means ± SD of four biological replicates (*n* = 4 plants). Five wild-type segregants (WT) obtained from lines *pSbUbi::schl-qsuB* #1, #2, #4, #5, and #7 were used as controls (*n* = 5 plants).

## Materials and Methods

### Generation of QsuB Transgenic Corn Lines

A *pSbUbi::schl*-*qsuB* expression cassette used previously to express *qsuB* in sorghum (Tian et al., 2022) was assembled into the PMS074 vector with a glufosinate-ammonium plant selectable marker using the jStack cloning method.^33^ The construct was introduced in the corn inbred line B104 by Phyto Regeneration Lab (Thousand Oaks, CA) via *Agrobacterium*-mediated transformation to generate glufosinate-resistant independent lines. T0 primary transformants with a single-copy event were identified by TaqMan PCR assays as previously described^34^ and self-pollinated manually. In the T1 and T2 generations, wild-type segregants and transgenic plants were identified by PCR using the *qsuB*-specific oligonucleotides 5′- TCTCCGATTGGTCTCGTGCG-3′ (forward) and 5′-GCAACCGGACAGCGTCATTT-3′ (reverse).

### Plant Growth Conditions

Plants were grown in a Sunshine Growing Mix #4 (Sun Gro) with two-gallon pots (T0 and T1 generations) or three-gallon pots (T2 generation) at the UC Berkeley greenhouse Oxford facility with minimum temperature set at 22 °C. One tablespoon of Osmocote plus 15–9–12 was added to the soil biweekly until the VT tasseling stage. The plants were self-pollinated manually. Watering was stopped at the end of the growing period and pots containing plants were allowed to dry for another 2 weeks. Ears were removed and mature seeds were collected. For analyses, plants without roots were harvested, further dried in an oven at 50 °C for 2 weeks, and subsequently ground using a knife mill equipped with a 2 mm mesh (Model 4 Wiley Mill, Thomas Scientific). Samples were additionally ground into a fine powder using a ball mill (Mixer Mill MM 400, Retsch Inc.) with stainless-steel balls for metabolite and cell wall composition analyses.

### Metabolite Extraction and Quantification

Metabolites were extracted from ball-milled biomass samples using 80% (v/v) methanol:water and subsequent acid-hydrolysis as previously described.^29^ DHBA and vanillate were analyzed in hydrolyzed methanol extracts using high-performance liquid chromatography (HPLC), electrospray ionization (ESI), and quadrupole time-of-flight (QTOF) mass spectrometry (MS) as previously described.^35^ Quantification was performed using calibration curves of standard solutions prepared with authentic compounds purchased from Sigma-Aldrich.

### Cell Wall Composition Analysis

The sequential extraction of ball-milled biomass (1 g) and the quantification of acid-insoluble lignin, acid-soluble lignin (absorbance at 320 nm), and monosaccharides (glucose, xylose, and arabinose) in the resulting cell wall residues (CWR) was conducted following the NREL standard laboratory analytical procedures.^36^ Cell-wall-bound aromatics were released from 20 mg of CWR via mild alkaline hydrolysis using a 2N sodium hydroxide solution as previously described.^37^ Ferulate, *p*-coumarate, DHBA, and vanillate were quantified using HPLC-ESI-QTOF-MS analysis.^35^

### Saccharification

Extracted biomass was used for cell wall saccharification assays. Dilute alkali (0.25% w/v sodium hydroxide) was used for biomass pretreatment and the enzyme mixture Ctec3 (Novozymes) was used for polysaccharide hydrolysis. The procedure and measurement of released monosaccharides were performed as previously described.^38^

### Two-Dimensional Nuclear Magnetic Resonance (2D-NMR) Spectroscopy

Samples consisted of a pool of biomass from five plants (2 g each) for each corn line. Before isolating the cellulolytic enzyme lignin (CEL) preparation, the samples were successively Soxhlet-extracted with acetone, methanol, and water (8 h each) to remove all extractives. CEL was then isolated by enzymatic hydrolysis of polysaccharides following the method described by Chang et al. (1975).^39^ We used Cellulysin (Calbiochem-Behring Corp.), a crude cellulase preparation from *Trichoderma viride* containing both cellulase and hemicellulase activities, with an activity of ≥ 10,000 units/g (based on reducing sugars released from filter paper at 40 °C, pH 4). Extractive-free, ball-milled material (300 mg) was suspended in 30 mL of 20 mM sodium acetate buffer (pH 5.0) in a 50 mL centrifuge tube. Cellulysin (40 mg) was added, and the mixture incubated at 30 °C for 48 h. Solids were recovered by centrifugation (8000 rpm, 4 °C, 20 min), and the hydrolysis step was repeated twice using fresh buffer and enzyme. The final lignin residue was collected by filtration, washed with water, and lyophilized.

For 2D-NMR analyses, around 50 mg of CEL were dissolved in 0.6 mL of DMSO-*d*_6_. 2D HSQC NMR spectra were recorded at 300 K on a Bruker AVANCE III 500 MHz instrument equipped with a cryogenically cooled 5 mm TCI gradient probe with inverse geometry, at the NMR facilities of the General Research Services of the University of Seville (SGI-CITIUS). The HSQC experiments were carried out using the Bruker standard pulse programs “hsqcetgpsisp2.2” and the following parameters: spectra were acquired from 10 to 0 ppm in F2 (^1^H) using 1000 data points for an acquisition time of 100 ms, an interscan delay of 1 s, and from 200 to 0 ppm in F1 (^13^C) using 256 increments of 32 scan, for a total experiment time of 2 h 34 min. The ^1^ *J*_CH_ used was 145 Hz. Processing used typical matched Gaussian apodization in ^1^H and a squared cosine bell in ^13^C. The central solvent peak was used as an internal reference (δ_C_/δ_H_ 39.5/2.49). 2D-NMR cross-signals were assigned by literature comparison.^40^ A semiquantitative analysis of the HSQC signals was performed using the Bruker’s Topspin 3.1 processing software. In the aromatic/unsaturated region, the content of H-, G- and S-lignin units was estimated using the correlation signals of H_2,6_, G_2_, and S_2,6_, respectively; the abundance of the different *p*-hydroxycinnamates was estimated using the signals for *p*CA_2,6_ and FA_2_; and the content of tricin was estimated using the signal for T_6_. As signals H_2,6_, S_2,6_ and *p*CA_2,6_ involve two proton–carbon pairs, their volume integrals were halved.

### Aromatic Extraction via Alkaline Pretreatment

Leaf and stem biomass from wild type and *pSbUbi::qsuB* #1 lines were milled (Retsch MM400) in a 50 mL stainless steel jar with one 15 mm stainless steel ball-bearing, shaking at 30 Hz for 3 min to obtain a fine powder. Milled biomass (2 g) was mixed with sodium hydroxide (1% NaOH, 20 mL) in sealed 125 mL Erlenmeyer flasks, treated for 90 min at 90 °C in an oil bath, and placed on ice for 10 min immediately after. The biomass and aqueous phase were separated by centrifugation (4300 rcf for 15 min) and the supernatant was recovered. The biomass solids were washed three times with distilled deionized water (20 mL, 15 mL, and 15 mL) and the washes collected by centrifugation. The supernatant and washes were combined, adjusted to pH 7.0 using 1 M hydrochloric acid, and subjected to ultracentrifugation (48,400 rcf, for 1 h at 4 °C) to remove any additional insoluble material. The supernatant solution constituted the alkaline pretreatment liquor (APL) used for PDC production. An acid-hydrolysis was performed on APL aliquots to release the aglycone forms of several aromatics and enable quantification. These include DHBA, 4-hydroxybenzoate, gallate, vanillate, syringate, 3-methylgallate, and 4-hydroxybenzaldehyde. The quantitative analysis of aromatic aglycones as well as free *p*-coumarate and ferulate in the APL was accomplished with a Shimadzu triple-quadrupole liquid chromatography–mass spectrometer (LC-MS, Nexera XR HPLC-8045 MS/MS). The mobile phase was a binary gradient that consisted of a mixture of water containing 0.1% formic acid (solvent A) and methanol (solvent B). The stationary phase was a Kinetex F5 column (Phenomenex, 2.6 μm pore size, 2.1 mm ID, 150 mm length, P/N: 00F-4723-AN). All compounds were detected by multiple-reaction-monitoring (MRM) and quantified using the strongest MRM transition.^41^

### Microbial Production of 2-Pyrone-4,6-dicarboxylate

A strain of *Novosphingobium aromaticivorans* DSM12444 engineered to produce PDC from aromatic phenolics was employed.^42^ The strain has deletions in genes encoding LigI (SARO_RS14300), DesC (SARO_RS14525), and DesD (SARO_RS14530) in the aromatic catabolic pathway to enable PDC accumulation from aromatics. The APL obtained from corn biomass was supplemented with glucose and ammonium sulfate (1 g/L each, final concentration) and filtered using 0.2 μm sterile poly­(ether sulfone) membrane filters. For PDC production, 10 mL aliquots of this solution were inoculated with 2 mL aliquots of an overnight culture of the engineered *N. aromaticivorans* strain grown at 30 °C (200 rpm) in standard mineral base media^43^ supplemented with glucose (3 g/L). Glucose supplementation is needed to provide a nonaromatic growth substrate since the aromatic catabolic pathways are truncated. After a 48-h growth period, 1 mL samples were collected and centrifuged at 3,300 rcf for 6 min to recover the supernatant, which was filtered for the quantification of aromatics and PDC using the LC-MS method described above.

## Results and Discussion

### Generation of Corn Expressing QsuB

A construct consisting of a *qsuB* open reading frame preceded with a sequence encoding a plastid-targeting peptide and placed downstream the promoter of a sorghum polyubiquitin gene was built for *Agrobacterium*-mediated corn transformation (Figure 1b). Seven primary T0 transformants resistant to glufosinate and containing a single copy of the *qsuB* transgene were identified by TaqMan real-time PCR assays and self-pollinated manually (data not shown). For each independent line, wild-type segregants and transgenic plants were identified by PCR in the T1 generation, self-pollinated manually, and grown to full maturity. The measurement of total leaf and stem dry weight showed that lines *pSbUbi:schl-qsuB* #3, #6, and #7 had significant reduction of biomass yield compared to wild-type controls (Table S1). Methanol-soluble metabolites were extracted from stem and leaf biomass and the methanolic extracts were acid-hydrolyzed to quantify total DHBA using HPLC-ESI-QTOF-MS analysis. Compared to wild-type plants, the QsuB transgenic lines showed large increases of DHBA content (up to 800-fold), which ranged between 22.5 and 28.7 mg/g dry weight (Figure 1c). These DHBA titers are an order of magnitude higher than those measured in switchgrass and sorghum lines transformed with the same *qsuB* gene.^30,32^ Moreover, the amount of vanillate was also increased in the QsuB corn lines, ranging between 1.2 and 2.2 mg/g dry weight, which represents 25- to 44-fold increases compared to control plants (Figure 1c). This result shows that native unknown *O*-methyltransferases convert a portion of the DHBA into vanillate. The analysis of nonhydrolyzed extracts revealed LC-MS peaks that display a primary molecular ion with a mass-to-charge ratio corresponding to that of DHBA glucose conjugates (Figure S1). These observations indicate that unknown UDP-glucosyltransferases glucosylate DHBA, leading to the formation of glucoside forms presumably stored in vacuoles. Lines *pSbUbi::schl-qsuB* #1, #2, and #7 were grown in the T2 generation for additional yield and growth measurements (Figure S2). The data showed that all three lines were similar to their respective wild-type segregants regarding the number of internodes, ear internode width, 100-seed weight, and tassel length. Lines #1 and #2 had a slightly longer ear leaf. However, lines #1 and #7 had slightly shorter ear internodes and line #7 had a smaller ear. Although statistically not significant, plant height at maturity was reduced by 10% in line #7, while the other two lines had similar height compared to their wild-type control. In terms of biomass yield, no significant change could be observed between the transgenic lines and wild type, except for line #7 that showed a 13% reduction in leaf dry weight (Figure S2). Overall, the data indicate that QsuB corn lines #1 and #2 display no major growth defects or yield penalty in the greenhouse.

Corn stover consists on average of cobs (15%), husks and shanks (10%), leaves (20%), and stems (60%).^44^ The analysis of acid-hydrolyzed metabolite extracts in these different plant parts in line *pSbUbi::schl-qsuB* #1 in the T2 generation showed higher DHBA content in the stem (37.6 mg/g dry weight) compared to the cob, leaves, and husks and shank (19.5–23.5 mg/g dry weight), possibly as a result of higher QsuB expression or larger pools of 3-dehydroshikimate in the stem (Figure S3). Analysis of nonhydrolyzed metabolite extracts revealed that free DHBA and vanillate were present in low amounts, accounting for no more than 2.5% and 4.5% of the total DHBA and vanillate, respectively, measured in hydrolyzed samples (data not shown). These findings indicate efficient glycosylation of both DHBA and vanillate in the various plant parts analyzed and suggest that the vacuole is the primary subcellular compartment for the localization of these two overproduced aromatic compounds.

### Cell Wall Composition Analysis and Saccharification Efficiency

The main cell wall components were measured in extractive-free biomass from total leaves and stems. Using the Klason method, lines *pSbUbi::schl-qsuB* #1, #3, #5, and #6 showed small reductions of acid-insoluble lignin in the range of 6–12% compared to wild-type controls, whereas acid-soluble lignin was increased in all the transgenic lines by 12–38% (Table 1). Reductions of acid-insoluble lignin have been measured previously in *Arabidopsis*, switchgrass, and poplar lines expressing QsuB,^29,30,45^ whereas no changes were observed in QsuB sorghum lines.^32^ The reduction of lignin occasionally observed in QsuB-engineered crops has been attributed to a reduction of the pool of shikimate used by the lignin biosynthetic enzyme hydroxycinnamoyl-CoA:shikimate hydroxycinnamoyltransferase.^46^ The amount of glucose was reduced by 12% and 7% in *pSbUbi::schl-qsuB* #1 and #6, indicating small reductions of cellulose in these two lines. Most of the transgenic lines had similar xylose content compared to wild-type controls, but the amount of arabinose was increased by 22–43%, which suggests a higher degree of arabinose substitution on xylan. Simple aromatics were released from cell walls by mild alkaline hydrolysis and quantified. The amount of *p*-coumarate was significantly reduced by 24–28% in four transgenic lines (#1, #3, #5, and #6) whereas ferulate was unchanged compared to the wild type. The observed reduction of *p*-coumarate is consistent with the reduction of lignin since most *p*-coumarate groups are found ester-linked to lignin.^47^ DHBA and VA released with this method were increased in all the transgenic lines by 7–9-fold and by 3–5-fold, respectively (Table 1). The cell wall alkaline hydrolysates from the transgenic lines also showed higher content of DHBA glucosides compared to the wild type (Figure S4).

**Table 1:** Chemical Composition of Cell Wall Residues (CWR) Obtained from Biomass of Wild Type and *pSbUbi::schl-qsuB* Lines^a^

	glucose	xylose	arabinose	acid-insoluble lignin	acid-soluble lignin	*p*-coumarate	ferulate	**DHBA** ^b^	vanillate	ash
wild type	369.4 (5.4)	204.6 (3.8)	28.1 (0.5)	148.6 (3.4)	17.3 (0.6)	21.2 (1.0)	5.1 (0.4)	10.6 (0.7)	0.3 (0.0)	35.9 (3.7)
*qsuB* #1	326.1 (12.4)*	155.4 (7.3)*	15.2 (0.3)*	139.6 (2.1)*	19.4 (0.2)*	16.0 (0.9)*	4.6 (0.4)	72.3 (7.8)*	0.9 (0.1)*	39.3 (5.8)
*qsuB* #2	354.6 (9.9)	192.1 (3.9)	34.3 (0.4)*	155.8 (4.0)	20.5 (0.9)*	19.4 (0.7)	4.4 (0.1)	92.8 (6.6)*	1.0 (0.1)*	32.8 (4.6)
*qsuB* #3	368.3 (1.7)	207.9 (3.0)	35.7 (0.7)*	130.3 (3.9)*	23.4 (0.4)*	15.2 (0.3)*	4,7 (0.2)	82.6 (2.2)*	1.0 (0.2)*	39.3 (1.3)
*qsuB* #4	380.1 (4.2)	203.1 (2.0)	34.6 (0.7)*	157.4 (5.0)	23.1 (0.4)*	20.8 (0.6)	5.0 (0.1)	86.3 (6.5)*	1.1 (0.1)*	31.0 (1.1)
*qsuB* #5	375.0 (4.2)	207.6 (1.2)	37.0 (0.5)*	134.4 (3.1)*	22.4 (0.6)*	17.7 (0.6)*	5.3 (0.1)	76.6 (3.3)*	0.8 (0.1)*	37.8 (1.0)
*qsuB* #6	342.8 (6.1)*	201.6 (4.2)	35.5 (2.3)*	132.6 (4.4)*	23.8 (0.4)*	16.1 (0.5)*	4.7 (0.2)	75.4 (1.5)*	1.0 (0.1)*	40.5 (0.6)
*qsuB #7*	359.9 (8.7)	204.1 (4.2)	40.2 (0.8)*	141.9 (6.8)	22.3 (0.6)*	18.4 (1.5)	4.8 (0.2)	74.8 (3.6)*	1.4 (0.2)*	30.5 (6.5)

a Values are expressed in mg/g CWR. Numbers in brackets are the SE from four or five biological replicates (*n* = 4 or 5 plants). Values in bold with an asterisk indicate a significant difference from the wild type using the unpaired Student’s *t* test (*P < 0.05).

b DHBA values are in μg/g CWR.

Saccharification assays were conducted to evaluate the recalcitrance of cell walls to enzymatic degradation. After a mild alkaline pretreatment followed by digestion with an enzyme mixture containing cellulases and hemicellulases, cell walls from lines *pSbUbi::schl-qsuB* #1 and #6 showed improvements of saccharification efficiency compared to wild type (Figure 2). In these two lines, glucose release was enhanced by 20% and 25% and xylose released was enhanced by 40 and 22%, respectively, indicating reduced cell wall recalcitrance.

**Figure 2:**
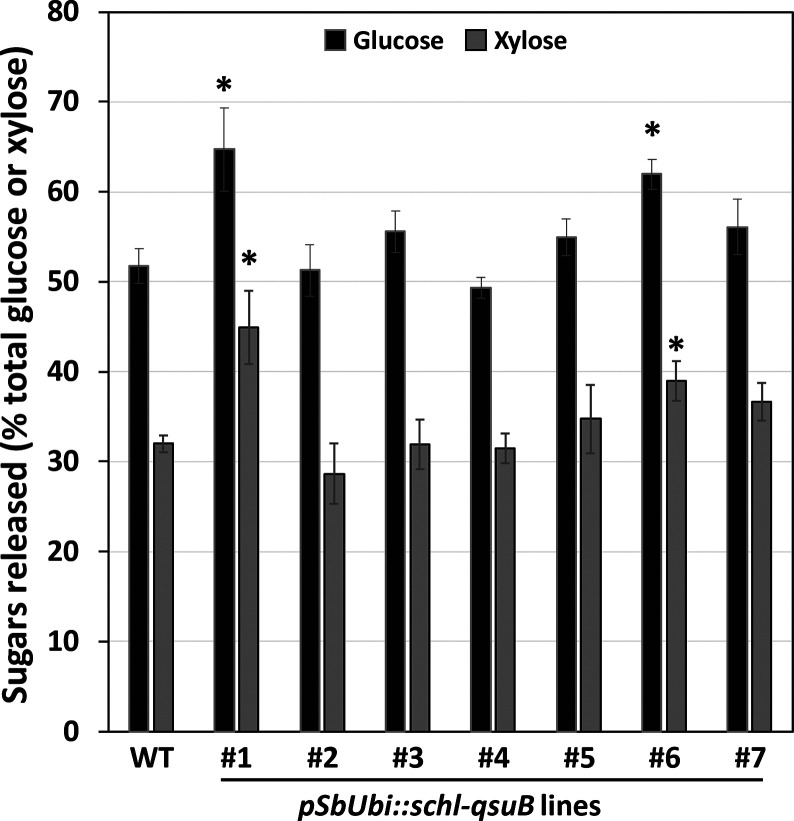
Saccharification efficiency of cell walls from wild type (WT) and QsuB corn. The values represent the amount of glucose and xylose released as a percentage of total glucose or xylose present in the cell wall samples, respectively. Cell walls were pretreated with dilute alkali and digested with a mixture of polysaccharidases for 72h. Values are means ± SD of four or five biological replicates (*n* = 4 or 5 plants). Asterisks indicate significant differences from the WT using the unpaired Student’s *t* test (*P < 0.05).

### Analysis of Lignin Monomer Composition

Lignin composition and the potential incorporation of DHBA into the QsuB-modified corn lignin were evaluated using 2D-HSQC-NMR spectroscopy. For this, cellulolytic enzyme lignin (CEL) preparations isolated from the wild type and lines *pSbUbi::schl-qsuB* #1 and #2 were subjected to NMR analysis. For more detailed characterization, the aliphatic–oxygenated (δ_C_/δ_H_ 50–90/2.5–5.8) and aromatic (δ_C_/δ_H_ 90–148/5.8–8.0) regions of the HSQC spectra were thoroughly analyzed. Figure 3 presents these spectral regions for the wild type (WT) and two QsuB-modified lines, along with the main lignin substructures identified. The HSQC spectrum of the CEL from the WT sample displayed characteristic lignin signals. In the aromatic region, distinct signals corresponding to syringyl (S), guaiacyl (G), and *p*-hydroxyphenyl (H) units were clearly observed, along with prominent signals from *p*-coumarates (*p*CA), ferulates (FA), and tricin (T). Quantitative analysis of the lignin composition indicated an H:G:S ratio of 3:40:57 (S/G ratio of 1.4), with *p*CA accounting for 67% relative to the total lignin units (H+G+S = 100), while FA and T each accounted for approximately 7%. In the aliphatic-oxygenated region, the spectrum was dominated by signals from β–*O*–4′ alkyl-aryl ether linkages (A), including γ-acylated counterparts (A′), along with signals from cinnamyl alcohol end-groups (I) and their γ-acylated counterparts (I′). In contrast, signals corresponding to other linkage types, such as β–5′ phenylcoumarans and β–β′ resinols, were barely detected. The CEL lignins from the QsuB lines #1 and #2 showed similar HSQC spectra to WT but with notable compositional differences. Both lines exhibited a higher proportion of S-units, leading to elevated S/G ratios, along with an increase in the content of H-units. Both transgenic lines showed an H:G:S composition of 5:26:69 (S/G ratio of 2.7). Although the physiological mechanism remains to be elucidated, increased lignin S/G ratios have also been reported in *Arabidopsis* and poplar lines expressing QsuB.^29,31^ While an elevated lignin S/G ratio has been occasionally linked to decreased biomass recalcitrance during enzymatic hydrolysis in certain plant species and following specific biomass pretreatments,^47^ line *pSbUbi::schl-qsuB* #2 did not exhibit an increase in sugar yield after dilute alkaline pretreatment and saccharification (Figure 2), indicating that the higher S/G ratio observed in this line does not influence cell wall recalcitrance under the conditions tested. In addition, the *p*CA content also increased substantially in both lines, reaching 80–81%, while T levels increased to 11%. The increase of *p*CA measured in CEL lignin samples is consistent with the observed higher S/G ratio as *p*CA ester groups mainly occur on S-units in lignin.^48^ In contrast, the FA content remained unchanged at 7%. In the aliphatic-oxygenated region, characteristic signals for β–*O*–4′ alkyl-aryl ether linkages (A) and cinnamyl alcohol end-groups (I), along with their γ-acylated counterparts (A′, I′), were clearly observed, similar to the spectrum of the WT control. However, and more important, no signals corresponding to benzodioxane (BD) structures—expected if DHBA were incorporated into lignin—could be detected in the QsuB lines. These results indicate that DHBA moieties were not incorporated into lignin under the conditions tested, suggesting that no transferase—including that involved in the transfer of *p*-coumarate^49^—has the capacity to couple DHBA with monolignols in corn. These results contrast with the previously observed incorporation of DHBA into lignin from QsuB poplar lines.^31^ This incorporation in poplar is possibly enabled by endogenous transferase(s) active on coenzyme A-activated benzoates, such as PHBMT1, which contributes to 4-hydroxybenzoate ester-linked moieties on lignin.^50^ The precise origin of DHBA and vanillate released from extractive-free corn biomass through mild alkaline hydrolysis is still unknown (Table 1). Similar to ferulate and *p*-coumarate, DHBA and vanillate in extracted biomass could be located on hemicellulose in the cell wall or be constituents of residual cutin.^51,52^

**Figure 3:**
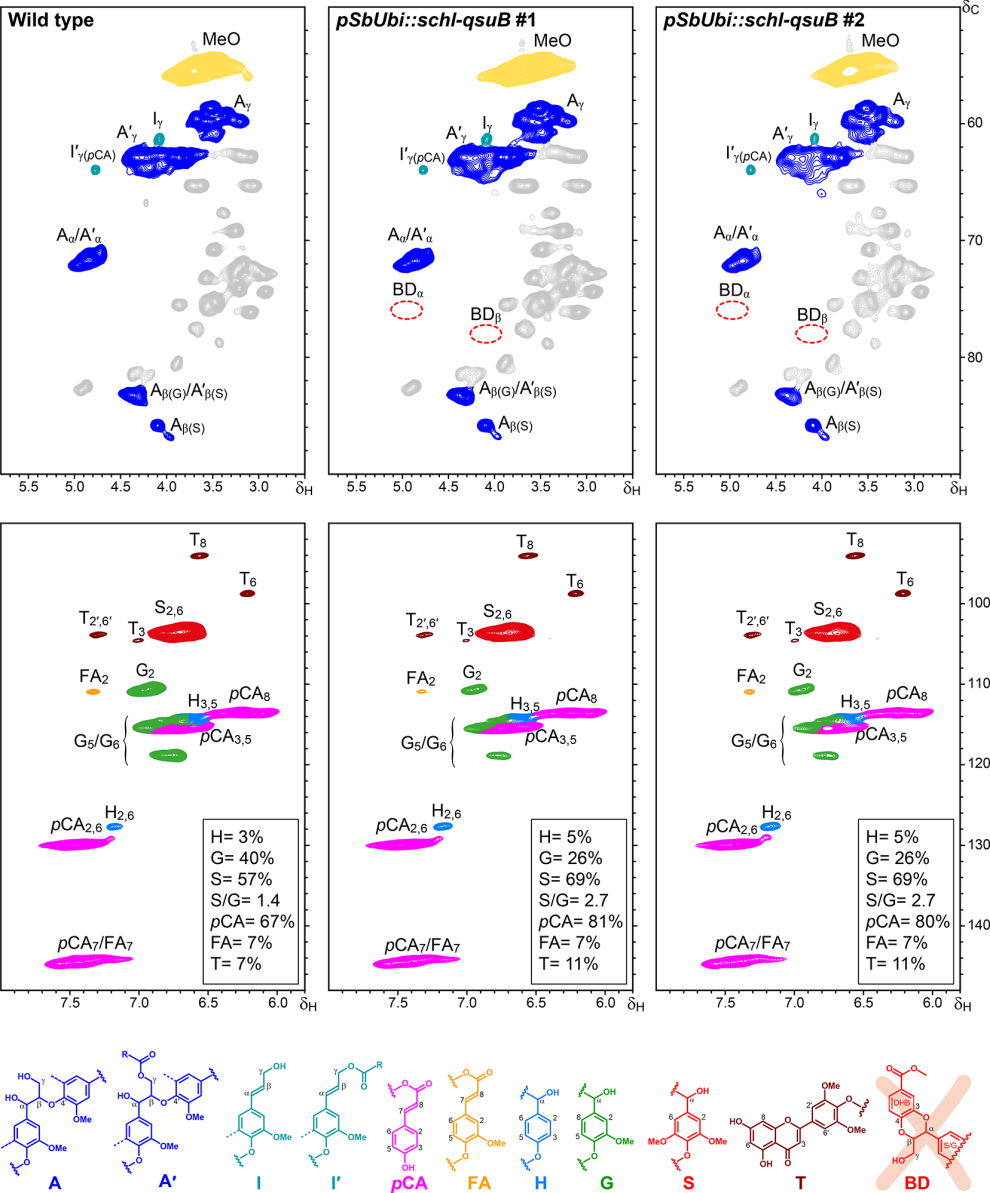
Aliphatic-oxygenated (top) and aromatic/unsaturated (bottom) sections of the HSQC spectra of the CEL isolated from the wild-type control and two QsuB-modified corn lines, *pSbUbi::schl-qsuB* #1 and #2. Main lignin substructures identified are β–*O*–4′ alkyl-aryl ethers (A), γ-acylated β–*O*–4′ alkyl-aryl ethers (A′), cinnamyl alcohol end-groups (I), γ-acylated cinnamyl alcohol end-groups (I′), *p*-coumarates (*p*CA), ferulates (FA), *p*-hydroxyphenyl units (H), guaiacyl units (G), syringyl units (S), and tricin (T). The benzodioxane (BD) structure which may result from the potential incorporation of DHBA, is also indicated. The structures are colored to match the assigned contours in the NMR spectra. The relative abundances of the different H-, G-, and S-lignin units, *p*CA, FA, and T, estimated from the integration of the signals in the HSQC spectra, are shown in appropriate boxes. The values of H-, G- and S-lignin units shown in the boxes are normalized to the total lignin units (H + G + S = 100%), and the content of the other lignin components (*p*CA, FA, T) are expressed relative to this total. Dashed red circles mark the regions in the spectra where the BD_α_ and BD_β_ signals are expected to appear.

### Aromatic Extraction and Biological Upgrading

Leaf and stem biomass from mature plants of corn line *pSbUbi::schl-qsuB* #2 and wild type was treated with a dilute sodium hydroxide solution to generate an alkaline pretreatment liquor (APL). The concentrations of aromatics present in the APL are shown in Figure 4a. Except for *p*-coumarate and ferulate, the amount of identified aromatics was higher in APL samples subjected to acid-hydrolysis, indicating the occurrence of these aromatics as glycoside conjugates. The analysis showed that the APL from the QsuB corn biomass is enriched 367-fold in DHBA compared to the APL from wild-type, making DHBA the most abundant aromatic. Moreover, vanillate, gallate, and 3-*O*-methylgallate could be detected only in the APL from QsuB biomass. In this APL, the levels of free DHBA (1.14 mM) and free vanillate (0.09 mM) accounted for 28% and 8%, respectively, of the total DHBA (4.04 mM) and vanillate (1.14 mM) measured in hydrolyzed samples. As expected, ferulate and *p*-coumarate were the two main aromatics in the APL from wild-type biomass, and *p*-coumarate content was reduced by 35% in the APL from the QsuB line tested. Regarding the content of less abundant aromatics, 4-hydroxybenzaldehyde was decreased by 23% in the APL from QsuB biomass, while 4-hydroxybenzoate and syringate were both increased by ∼ 30%. Previous in-depth analyses of APL components have shown that free monoaromatics typically constitute less than 5% of the total carbon, while hydroxyacids can account for approximately 10%. The remaining portion consists of unidentified lignin dimers, oligomers, and high molecular weight lignin.^53,54^ HPLC chromatograms of the APL samples showed the presence of unidentified compounds, but no other unknown free monoaromatics were detected at high concentrations (Figures S5–S6).

**Figure 4:**
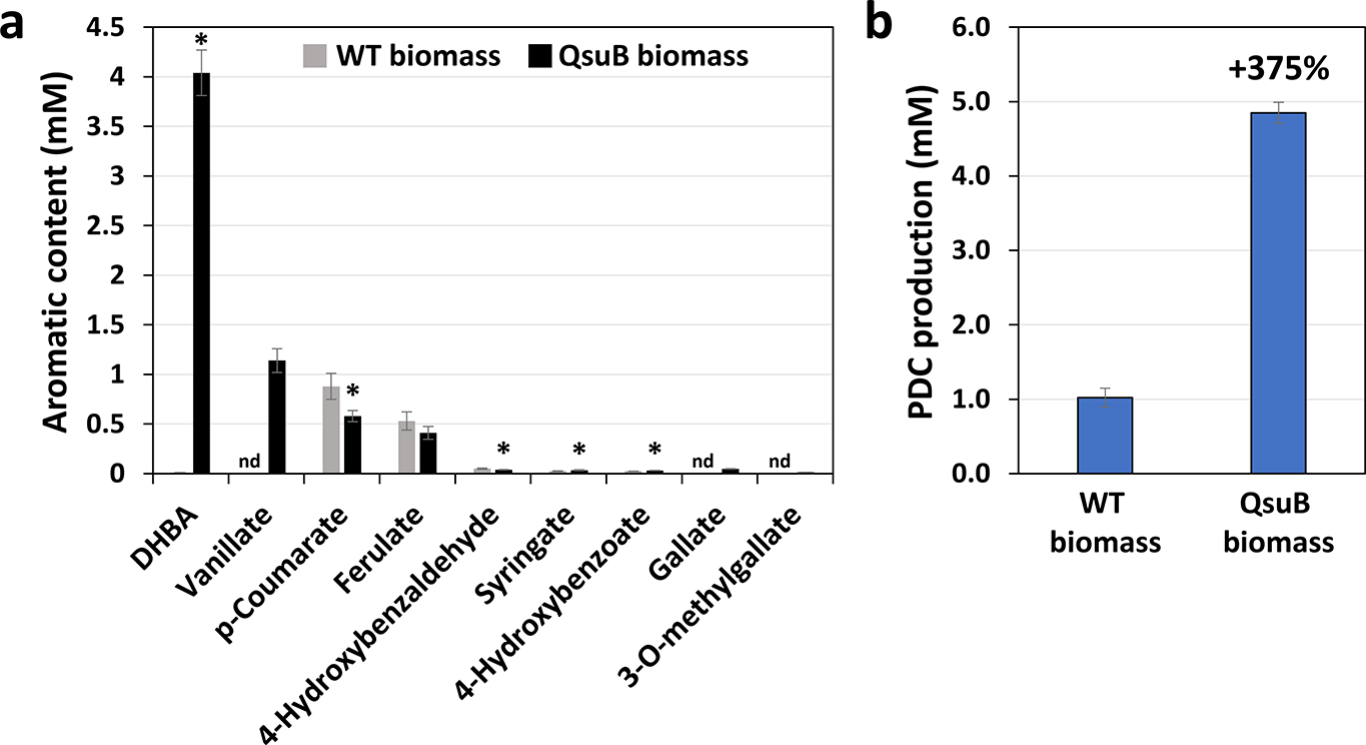
Composition of APL derived from corn biomass and PDC production by engineered *N. aromaticivorans.* (a) Aromatic content in APL obtained from wild-type (WT) and QsuB corn biomass. Values are means ± SD of three technical replicates. Benzoate and benzaldehyde levels were quantified in acid-hydrolyzed APL samples. Ferulate and *p*-coumarate values were obtained from nonhydrolyzed samples. Asterisks indicate significant differences from the WT using the unpaired Student’s *t* test (*P < 0.05). nd, not detected. (b) Amount of PDC produced after growing the engineered *N. aromaticivorans* strain for 48h on APL derived from WT or QsuB corn biomass. Values are means ± SD of three technical replicates.

For bioproduction, the APL samples from QsuB and wild-type corn stover were supplemented with glucose and ammonium sulfate, and inoculated into growth media with an engineered *N. aromaticivorans* strain that produces the polyester precursor 2-pyrone-4,6-dicarboxylate (PDC) from plant-derived aromatics.^42^ After a growth period of 48 h, all the free aromatics were no longer detectable in the culture medium, except for trace amounts of DHBA and syringate (<60 μM) (data not shown). The amount of PDC produced reached 4.9 mM (883 mg/L) in the cultures grown on APL derived from QsuB biomass, representing a 4.8-fold increase compared to the PDC titers achieved in the cultures grown on APL derived from wild-type biomass (Figure 4b). These results indicate that aromatics are taken up efficiently by *N. aromaticivorans* and converted into PDC via aromatic catabolic pathways.^11,55^ A PDC yield of 80% was obtained, based on the initial concentrations of *p*-coumarate (0.58 mM), ferulate (0.41 mM), total DHBA (4.04 mM), and vanillate (1.14 mM) found in the APL from QsuB biomass. The lack of stoichiometry could be due to *N*. *aromaticivorans* not being able to efficiently metabolize certain forms of conjugated aromatics, or the existence of alternative DHBA degradation pathways that divert aromatic carbon away from PDC production. DHBA represents a key intermediate in the catabolism of aromatic compounds, and while 4,5-extradiol DHBA catabolism through PDC has been established as the primary pathway,^42^ an alternative route involving DHBA decarboxylation to catechol has also been recently identified.^56^ The regulation of carbon flow from DHBA, either through or away from PDC, by *N. aromaticivorans* is currently under active investigation.^57,58^ It is conceivable that particular conjugated forms of DHBA might induce an uncharacterized metabolic response.

A portion of the aromatics occur as glycoside conjugates in the APL, which raises the question as to whether *N. aromaticivorans* hydrolyzes these conjugates before or after transporting them intracellularly. A transporter (PcaK) has been involved in the uptake of aromatics including DHBA, vanillate, 4-hydroxybenzoate, and syringate in *N. aromaticivorans*, but its capacity to transport aromatic glycosides remain to be determined.^59^ Several β-glucosidases from the CAZy glycoside hydrolase (GH) families 1 and 3 are known to be active on aromatic glucosides,^60^ and the *N. aromaticivorans* genome contains at least two and five genes encoding GH1 and GH3 enzymes, respectively (https://www.cazy.org/b350.html). For instance, SARO_RS02700 (GH1) and SARO_RS08655 (GH3) share 59% and 67% identity, respectively, with beta-glucosidases from *Sphingomonas paucimobilis* and *Novosphingobium* sp. GX9. These latter enzymes have demonstrated activity on aromatic glucosides, including *p*-nitrophenylglucoside, arbutin, salicin, and esculin.^61,62^

## Conclusions

Previous techno-economic analyses indicated that isolating compounds from bioenergy crops could improve the economics of advanced bioproducts made via fermentation of lignocellulose. For example, using sorghum as feedstocks, it has been modeled that accumulating DHBA at titers >0.3 dwt% in biomass could enable lower-cost biofuels because the revenue from DHBA extraction significantly surpasses the cost of extraction, which occurs before the downstream conversion of biomass to biofuel.^32^ Similarly, it has been calculated that isolating aromatic monomers (e.g., 4-hydroxybenzoate) produced in engineered crops at titers >3 dwt% can be cost-competitive compared to microbial fermentation routes, even if microbial platforms were to reach theoretical maximum yields.^63^ Therefore, the DHBA titer of 2.9 dwt% achieved in corn could potentially improve the economics of advanced bioproducts and deliver DHBA at a cost that competes with microbially produced DHBA if corn stover serves as a biorefinery feedstocks. We demonstrated efficient extraction of DHBA from engineered corn using aqueous methanol as solvent, which is due to DHBA accumulating as soluble glucosides in biomass extractives, rather than forming covalent linkages with cell walls. This is a key advantage of the current approach since chemicals produced in crops should accumulate as readily extractable forms that can be isolated from biomass while simultaneously preserving cell wall polysaccharides for downstream applications, such as fiber production or fermentation into advanced bioproducts.^64^ We also employed dilute alkali to extract both DHBA and cell-wall-bound ester-linked *p*-coumarate and ferulate for downstream valorization into PDC via biological funneling. Successfully, higher PDC titers were obtained with the hydrolysate from QsuB-modified corn biomass due to elevated DHBA and VA contents. Such DHBA-enriched aromatic streams could be employed for the production of a wide range of chemicals made by engineered microbial strains that use DHBA as central intermediate.^65,66^

Additional genetic modifications to the feedstock could enhance DHBA titers. Our previous research with *Arabidopsis* and tobacco showed that coexpressing QsuB with a bacterial feedback-insensitive 3-deoxy-d-arabinoheptulosonate 7-phosphate (DAHP) synthase—the first enzyme in the shikimate pathway—resulted in DHBA titers up to 350% higher than when QsuB was expressed alone,^67^ indicating an opportunity for stacking DAHP synthase with QsuB in corn. The next step toward advancing the in-planta production of DHBA involves field assessment of engineered corn to evaluate both stover and grain yields. The QsuB engineering approach conferred drought tolerance in *Arabidopsis*,^68^ and DHBA overproduction in switchgrass was shown to modulate plant microbiomes,^69^ but it remains to determine if these changes will also occur in corn and how they will affect growth and yield under natural environments.

## Supplementary Material



## Notes

Table of biomass yields in QsuB transgenic corn and wild-type controls (T1 generation); Figure of an UPLC-ESI-TOF MS chromatogram of DHBA glucose conjugates isolated from QsuB corn extracts; Figure of growth parameters in QsuB corn and wild-type controls (T2 generation); Figure of DHBA and vanillate contents in different parts of QsuB corn; Figure of the quantification of DHBA glucosides released from cell walls by alkaline hydrolysis; Figures of representative HPLC chromatograms of APL from corn biomass, before and after incubation with the PDC-producing *N. aromaticivorans* strain. (PDF)

## ABBREVIATIONS

AAA: aromatic amino acids
CWR: cell wall residue
DAHP: 3-deoxy-d-arabinoheptulosonate 7-phosphate
DHBA: protocatechuate
DW: dry weight
HPLC-ESI-QTOF-MS: high-performance liquid chromatography-electrospray ionization quadrupole time-of-flight mass spectrometry
2D NMR: two-dimensional nuclear magnetic resonance
