# Supplementary material for: Engineered Accumulation of Protocatechuate in Corn Biomass to Enhance Biomanufacturing
Source: ACS Sustain Chem Eng. 2025 Nov 11;13(46):20204–14. doi: 10.1021/acssuschemeng.5c09025 (PMC12648766; doi:10.1021/acssuschemeng.5c09025)
Supplement: Supplementary file 1 [file sc5c09025_si_001.pdf]

SUPPORTING INFORMATION

Engineered Accumulation of Protocatechuate in Corn Biomass to Enhance Biomanufacturing

Yang Tian[1,2], Bumkyu Kim[3,4,5], Irem Pamukçu[2], Emine Akyuz Turumtay[1,6], Alexis H. Tan[2], Victoria Saini[2], Ariana Irma Chavez[2], Anna Tang[2], Anna Z. Su[2], Edward E. K. Baidoo[1,6], Jorge Rencoret[7], José C. del Río[7], Timothy J. Donohue[4,5,8], Daniel R. Noguera[3,4,5], Aymerick Eudes[1,2*]

[1] Joint BioEnergy Institute, Emeryville, CA 94608, USA
[2] Environmental Genomics and Systems Biology Division, Lawrence Berkeley National Laboratory, Berkeley, CA 94720, USA
[3] Department of Civil and Environmental Engineering, University of Wisconsin-Madison, Madison, WI 53706, USA
[4] Wisconsin Energy Institute, University of Wisconsin-Madison, Madison, WI 53726, USA
[5] Great Lakes Bioenergy Research Center, University of Wisconsin-Madison, WI 53726, USA
[6] Biological Systems and Engineering Division, Lawrence Berkeley National Laboratory, Berkeley, CA 94720, USA
[7] Instituto de Recursos Naturales y Agrobiología de Sevilla, IRNAS-CSIC, Avenida de la Reina Mercedes, 10, 41012-Sevilla, Spain
[8] Department of Bacteriology, University of Wisconsin-Madison, WI 53706, USA

*Corresponding author: A.E. (ageudes@lbl.gov)

Number of pages: 8
Number of figures: 6
Number of tables: 1

- **Table S1:** Biomass yields from mature QsuB transgenic corn plants and wild-type controls in the T1 generation
- **Figure S1:** Representative UPLC-ESI-TOF MS chromatogram of DHBA glucose conjugates extracted from engineered corn biomass
- **Figure S2:** Growth parameters of lines *pSbUbi::schl-qsuB* #1, #2, and #7 and their respective wild-type segregants in the T2 generation
- **Figure S3:** DHBA and vanillate contents in different parts of line *pSbUbi::schl-qsuB* #1 in the T2 generation
- **Figure S4:** Quantification of DHBA glucosides released from cell walls by alkaline hydrolysis
- **Figure S5:** Representative HPLC chromatograms of APL from QsuB corn biomass, before and after incubation with the PDC-producing *N. aromaticivorans* strain.
- **Figure S5:** Representative HPLC chromatograms of APL from wild-type corn biomass, before and after incubation with the PDC-producing *N. aromaticivorans* strain.

Table S1. Biomass yields from mature QsuB transgenic corn plants and wild-type controls in the T1 generation. Total leaves and stems were harvested from fully mature plants. Values are means ±SD of four to eight biological replicates for the *pSbUbi::schl-qsuB* lines (n = 4–8 plants). Ten wild-type segregants obtained from the different *pSbUbi::schl-qsuB* lines were used as controls (n = 10 plants). Asterisks indicate significant differences from the wild type using the unpaired Student's t-test (*P < 0.05).

Line name	Dry weight (g)
Wild type	183.8 ± 13.3
pSbUbi::schl-qsuB #1	163.3 ± 15.3
pSbUbi::schl-qsuB #2	168.6 ± 14.4
pSbUbi::schl-qsuB #3	112.9 ± 8.7*
pSbUbi::schl-qsuB #4	205.1 ± 11.8
pSbUbi::schl-qsuB #5	152.7 ± 12.6
pSbUbi::schl-qsuB #6	111.1 ± 23.4*
pSbUbi::schl-qsuB #7	131.3 ± 10.9*

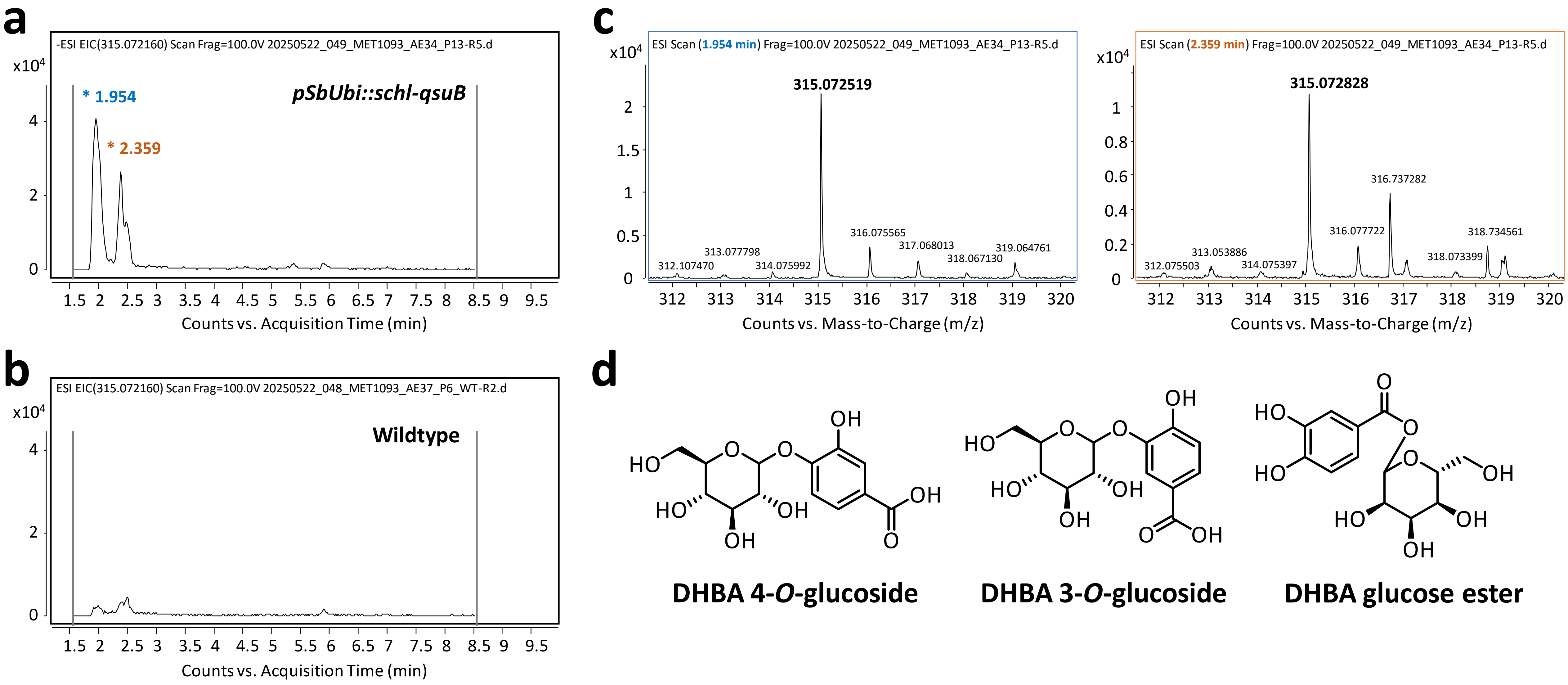

Figure S1: Representative UPLC-ESI-TOF MS chromatogram of DHBA glucose conjugates extracted from engineered corn biomass. (**a**) Transgenic line *pSbUbi::schl-qsuB* #7, (**b**) wild-type control, (**c**) mass spectra, and (**d**) putative chemical structures of glucosylated DHBA.

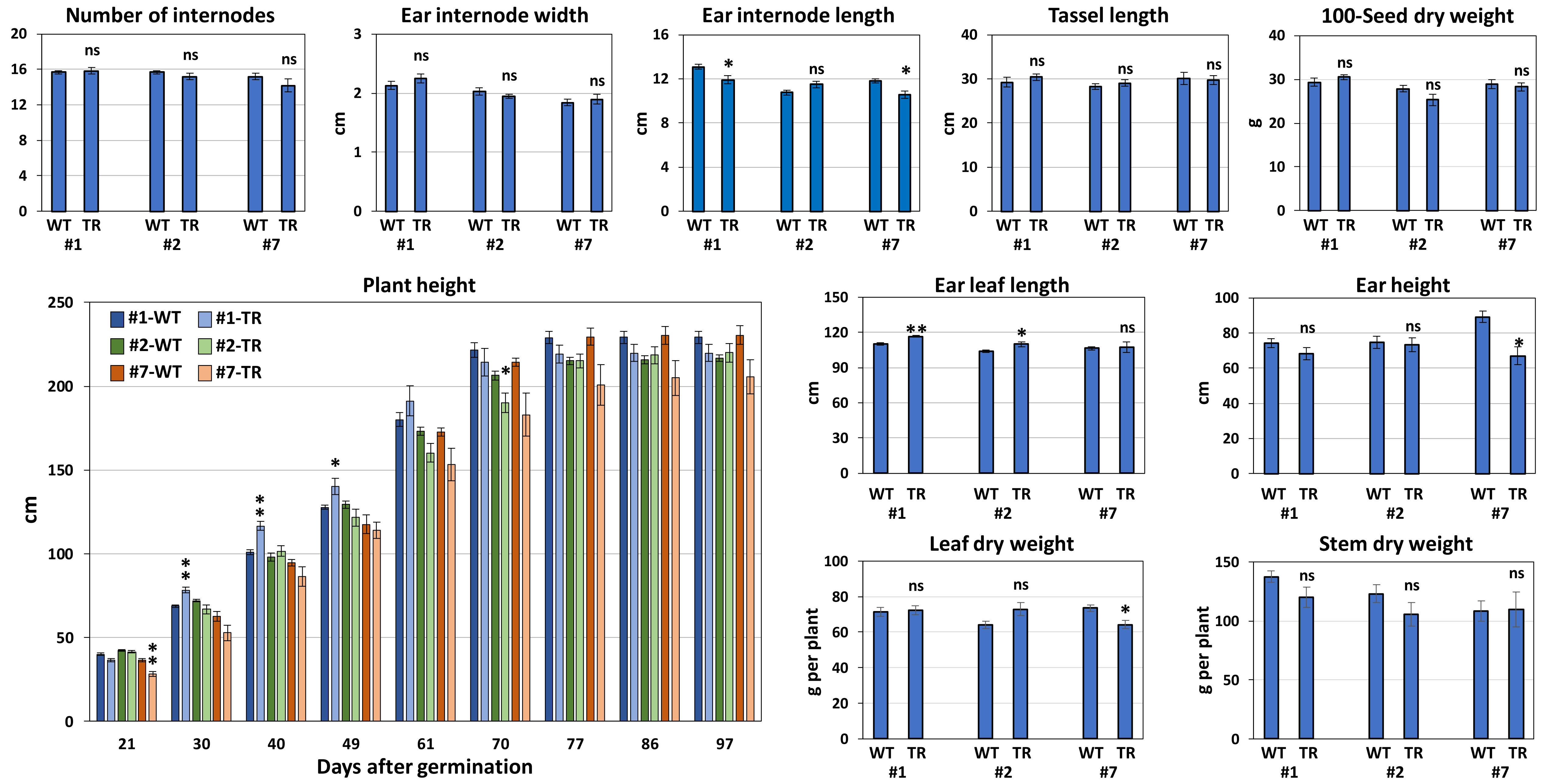

Figure S2: Growth parameters of lines *pSbUbi::schl-qsuB* #1, #2, and #7 and their respective wild-type segregants (WT) in the T2 generation. Plant height was measured at different time points after seed germination. Other parameters were measured on fully mature plants at the R6 reproductive stage. Values are means ±SD of five to six biological replicates (n = 5–6 plants). Asterisks indicate significant differences from the corresponding WT segregant using the unpaired Student's t-test ($*P < 0.05$, $**P < 0.005$). ns, not significant.

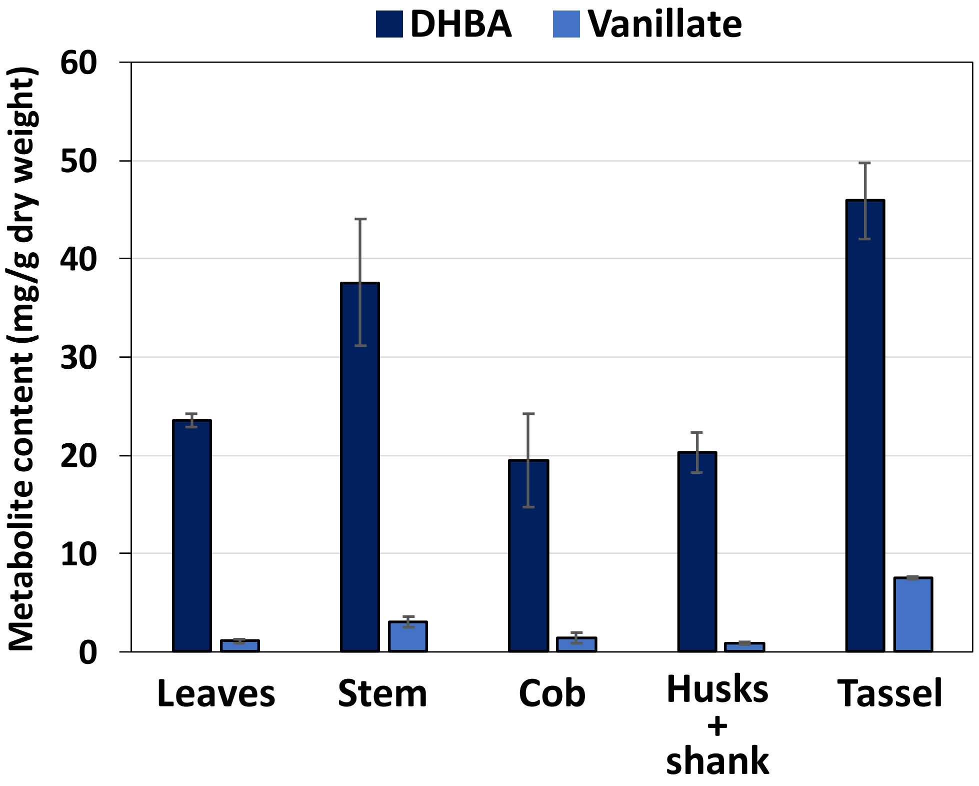

Figure S3: DHBA and vanillate contents in different parts of line *pSbUbi::schl-qsuB* #1 in the T2 generation. Values are means ±SD of four biological replicates (*n* = 4 plants).

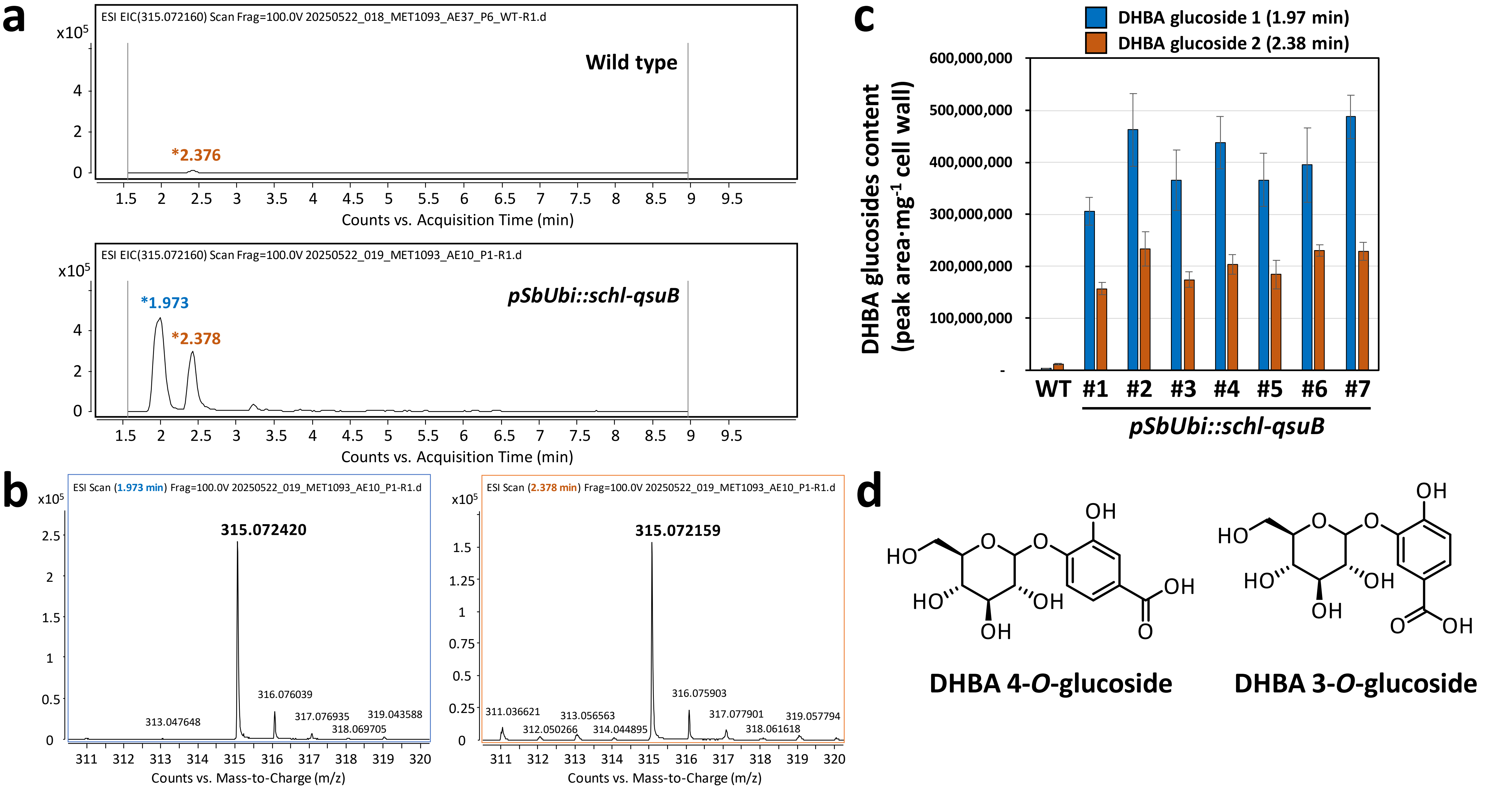

Figure S4: Quantification of DHBA glucosides released from cell walls after alkaline hydrolysis. Representative (**a**) UPLC-ESI-TOF MS chromatograms and (**b**) mass spectra of DHBA glucose conjugates extracted from wild type (top panel) and transgenic line *pSbUbi::schl-qsuB* #1 (bottom panel). (**c**) Relative content and (**d**) putative chemical structures of DHBA glucosides.

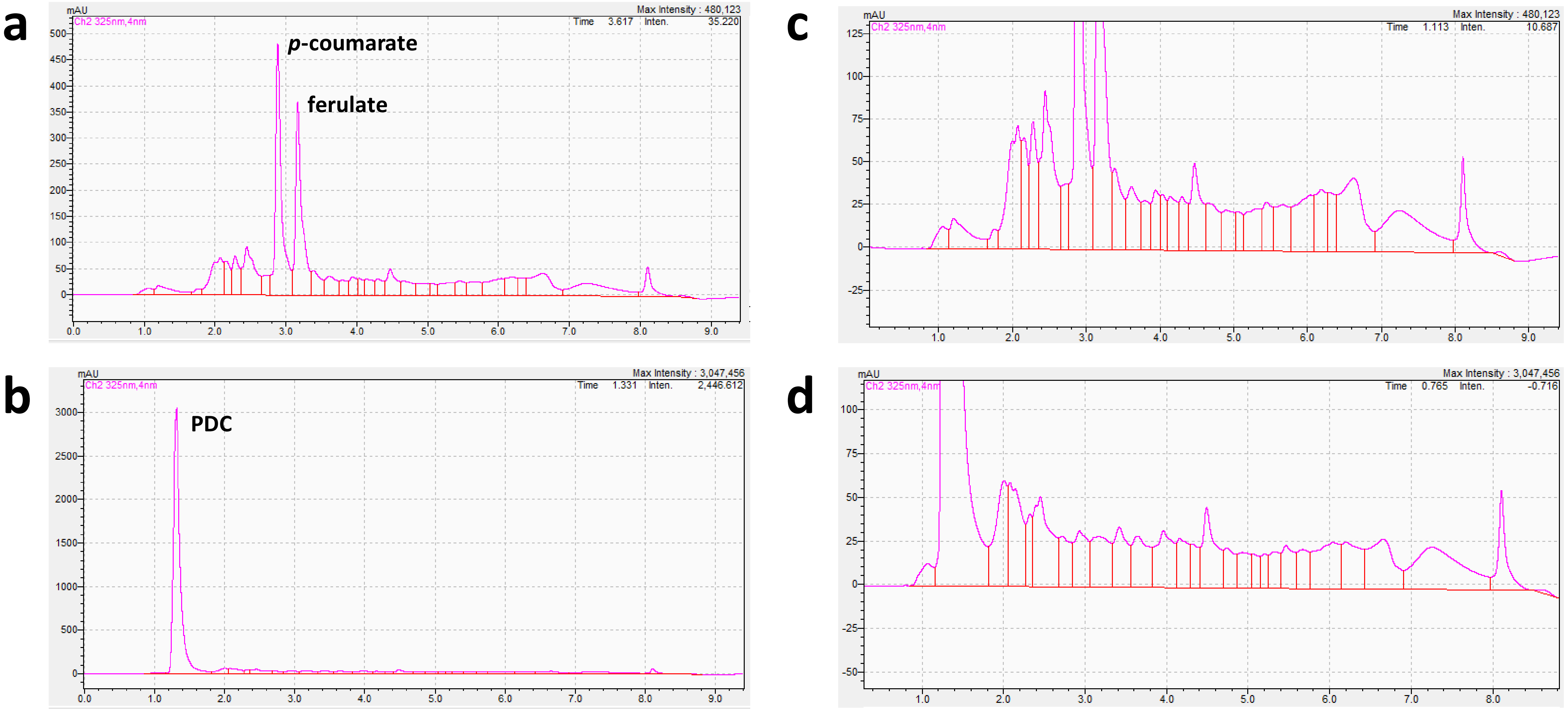

Figure S5: Representative HPLC chromatograms of APL from QsuB corn biomass, before and after incubation with the PDC-producing *N. aromaticivorans* strain. The chromatograms are shown for absorbance at 325 nm, which allows simultaneous visualization of the aromatic compounds and PDC. (**a**) Before microbial growth, (**b**) after microbial growth and PDC production. Panels (**c**) and (**d**) are zoomed in chromatograms of (a) and (b), showing the typical elevated baseline indicative of other unidentified compounds in the APL.

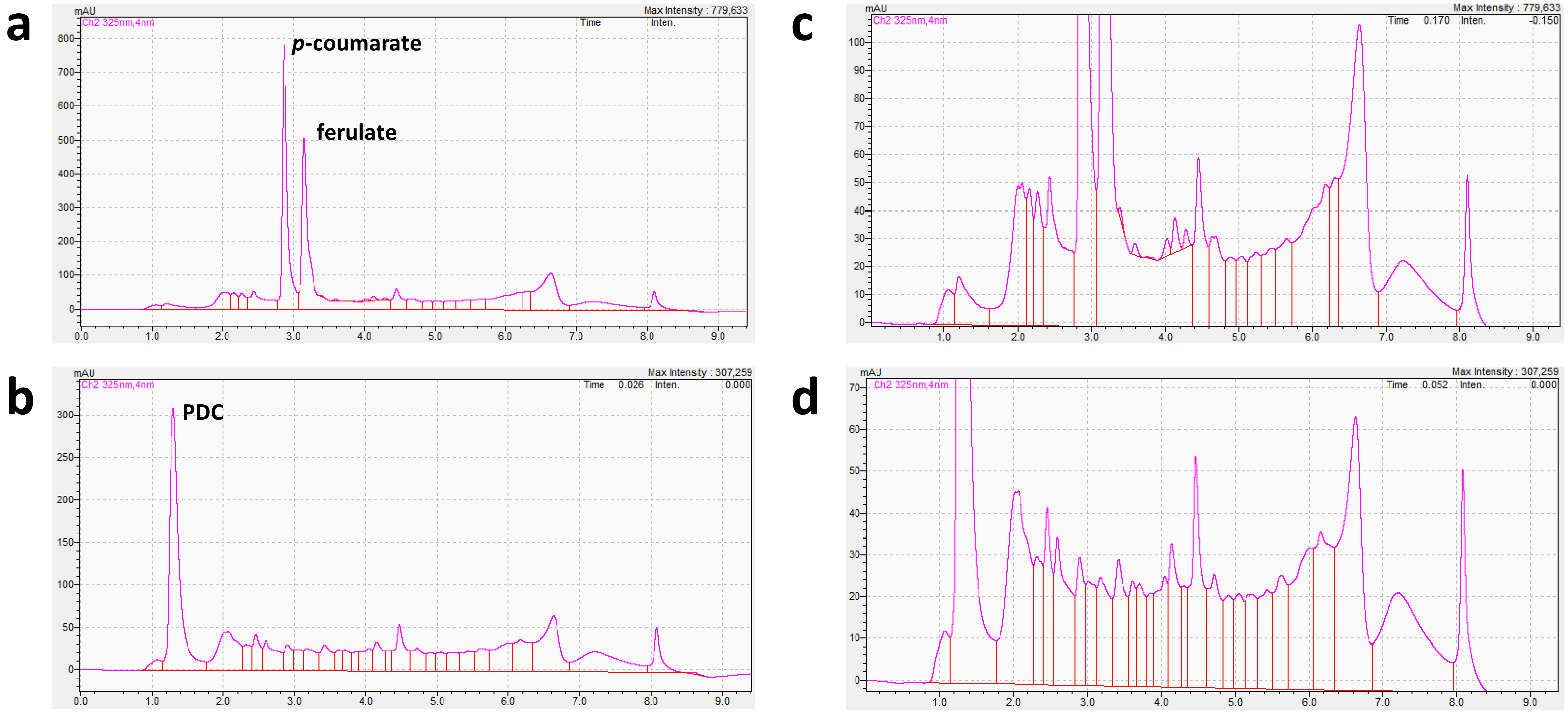

Figure S6: Representative HPLC chromatograms of APL from wild-type corn biomass, before and after incubation with the PDC-producing *N. aromaticivorans* strain. The chromatograms are shown for absorbance at 325 nm, which allows simultaneous visualization of the aromatic compounds and PDC. (**a**) Before microbial growth, (**b**) after microbial growth and PDC production. Panels (**c**) and (**d**) are zoomed in chromatograms of (a) and (b), showing the typical elevated baseline indicative of other unidentified compounds in the APL.
